# Supplementary material for: Elevated Serum Levels of IgG4 in Patients with Heart Failure with Reduced Ejection Fraction: A Prospective Controlled Study
Source: Biology (Basel). 2022 Aug 4;11(8):1168. doi: 10.3390/biology11081168 (PMC9404706; doi:10.3390/biology11081168)
Supplement: Supplementary file 1 [file biology-11-01168-s001.zip › biology-1625607-supplementary.pdf]

**Table S1.** Levels of markers in patients with ischemic DCM, idiopathic DCM and patients without DCM (controls).

| | **NT proBNP (pg/mL)** | ***P* value** |
|---|---|---|
| Ischemic vs Control | 931±1169 vs 36±34 | <0.01 |
| Idiopathic vs Control | 1183±2180 vs 36±34 | <0.01 |
| Ischemic vs Idiopathic | 931±1169 vs 1183±2180 | 0.49 |
| | **CRP (mcg/mL)** | ***P* value** |
| Ischemic vs Control | 78±31 vs 32±26 | <0.01 |
| Idiopathic vs Control | 82±59 vs 32±26 | <0.01 |
| Ischemic vs Idiopathic | 78±31 vs 82±59 | 0.28 |
| | **ST2 (pg/mL)** | ***P* value** |
| Ischemic vs Control | 717±617 vs 123±94 | <0.01 |
| Idiopathic vs Control | 464±353 vs 123±94 | <0.01 |
| Ischemic vs Idiopathic | 717±617 vs 464±353 | 0.01 |
| | **IgG4 (mg/dL)** | ***P* value** |
| Ischemic vs Control | 90±67 vs 50±29 | <0.01 |
| Idiopathic vs Control | 67±60 vs 50±29 | 0.08 |
| Ischemic vs Idiopathic | 90±67 vs 67±60 | 0.051 |

DCM – dilated cardiomyopathy, NT proBNP – amino-terminal pro-B-type natriuretic peptide, CRP – C-reactive peptide, ST2 –interleukin 1 soluble receptor, IgG4 – immunoglobulin gamma subclass 4.
